# The Key Role of Social Movements in Protecting the Health of People and the Planet: Comment on "Ensuring Global Health Equity in a Post-pandemic Economy"

**DOI:** 10.34172/ijhpm.2023.7757

**Published:** 2023-04-15

**Authors:** Chiara Bodini

**Affiliations:** ^1^Centre for International and Intercultural Health, University of Bologna, Bologna, Italy; ^2^People’s Health Movement

**Keywords:** Post-Pandemic Economy, Degrowth, Social Movements, Democracy

## Abstract

In his recent article, titled "Ensuring Global Health Equity in a Post-pandemic Economy," Ronald Labonté addresses a key challenge the world is facing, trying to ‘build back’ after the global crisis related to the COVID-19 pandemic. He explores and critically examines different policy options, from a more inclusive ‘stakeholder model’ of capitalism, to a greater role of states in shaping markets and investing in the protection of health and the environment, to more radical options that propose to reframe the capitalist mantra of growth and look at different ways to value and center our societies around what really matters most to protect life. Social movements are key players in such transformation, however the political space they move in is progressively shrinking.

 In his recent article, Ronald Labonté addresses a key challenge the world is facing, trying to ‘build back’ after the global crisis related to the COVID-19 pandemic.^1^ The author’s central argument is that, if we do not want to go back to a situation that led to such crisis (both in terms of creating the conditions for a global pandemic, and of crippling the possibilities of a coordinated and just global response), we have to reconsider the premises of our whole economic and social system. This assumption is apparently shared by many, from global institutions to states, to civil society organizations and social movements. The key question that the paper tries to address, therefore, is what sort of post-pandemic economic world we should strive to achieve, if we assume that the collective goals to pursue are health equity and environmental sustainability.

 In order to answer this question, the author critically examines a set of options that are being considered by states and other institutions in order to shape a post-pandemic economy different from the one we know. In his journey he is accompanied, and somehow informed, by the reflections of three economists he interviewed and who have given considerable thoughts to the issue: Walden Bello, Tim Jackson, and Jayati Ghosh.

 The author’s starting point is a lucid analysis of the ‘existential (health) crises’ that were there before the pandemic: rising inequalities (wealth, income, resources), ecological collapse (climate change and more), and migration (within and across borders). According to his view, and to the economists he interviewed, the current economic system is to blame for allowing a minority of the world’s population (the billionaire class) to continue increasing its wealth as the overwhelming majority becomes poorer. Moreover, the myth of capitalist growth economy — resting on levels of material consumption that are inequitable and unsustainable for a finite world — is responsible for a degree of environmental degradation that threatens life on our planet.

 Facing such a dire situation with a determination to look for alternative paths, the author takes into consideration different policy options, that have been mentioned in relation to the post-pandemic recovery by a number of countries, mainly in the Global North, and international institutions.

 A first option, promoted by the World Economic Forum in its call for a ‘Great Reset,’ is a shift from the current ‘shareholder’ to a new form of ‘stakeholder’ capitalism, where everyone, and not just shareholders, may have a stake in the system’s benefits. Investing in activities aligned with the Sustainable Development Goals, for instance, may at once produce profits and generate benefits for the people.^2^ However, as pointed out by critics of this model, profit is the driving force behind such investments, and — without robust systems being put in place to ensure accountability and redistribution — the gains they generate will continue to be unequally distributed. Moreover, as illustrated in a recent study on the flaws of so-called ‘multistakeholder capitalism,’ such model will likely strengthen the role of the private sector in global governance, reducing the accountability of governments and multilateral institutions.^3^

 A second option sees an increased role of the state in mitigating the inequalities generated by the market, something that — although theoretically denied by the neoliberal doctrine — clearly happened during the 2008 economic crisis. In that case, public money was used to bail out banks ‘too big to fail,’ but this was quickly followed by a round of austerity measures aiming to reduce public expenditure and government debt. Citizens paid the price for such measures in terms of weakened welfare state. For instance, a consequence of such policies was the dismantling of healthcare systems in many European countries, something that had a visible impact on the (un)preparedness towards the COVID-19 pandemic. This in turn made manifest the need for new and greater public investments in health and social protection, with governments of the United States and Europe committing to new plans to increase public spending and at the same time protecting the environment. However, these efforts seem insufficient in their scale, and are now additionally threatened by the energy crisis linked to the war in Ukraine.

 A third option considered in the paper looks at the possibilities to increase tax revenues, by expanding the fiscal space (that has progressively been shrunk in the past decades), contrasting tax heavens, and introducing taxes on financial transactions. Together with bold monetary policies and a reform of the International Monetary Fund loan system, more redistributive tax systems may have the potential to recapture public wealth for public good purposes and allocate it equitably. However, doing so requires a quite dramatic shift in how states see their role in the economy, as entities whose role is to shape markets and make them work towards democratically determined health, social, and environmental goals.

 Finally, the author turns to what he seems to consider the most promising options, centered around a deep rethinking of the current economic system. In a spectrum of discourses, such options go under the names of ‘degrowth,’ ‘fair growth,’ or ‘post growth.’ In short, they all postulate the need to reduce aggregate global consumption levels to avoid catastrophic ecosystem collapse. Given that consumption patterns have historically been very unequal between the Global North and the Global South, there is one part of the planet who needs to significantly reduce the amount of resources it consumes, while for still a large part of the world’s population growth is indeed important to achieve healthy life expectancies. According to these perspectives, it is not only important to reduce and redistribute consumption, but also to center our economies around the common good, which includes protecting our ecosystem and valuing the occupations that help us to live better. The ecological economist Tim Jackson, interviewed by the author, speaks about ‘care economy,’ centered around engagement, attention, and time in the service of each other.

 In order to achieve a transformation towards an economic system that is not centered around growth, a substantial change in the role of governments is needed, towards a system in which the state has and exercises the power to regulate (shape) markets, increase its revenues to invest in health and social protection, support an economy centered around the protection and promotion of human and non-human life. This brings the author to a set of conclusive considerations, acknowledging that – in the words of Walden Bello – “we can’t leave it just to the politicians.” The issue of democracy and government accountability, particularly facing the rise of authoritarian regimes in many parts of the world, becomes central if states have to shift towards policies that truly protect their citizens. The paper ends by mentioning social movements (global climate strikes, Black Lives Matter, *buen vivir* and peasant’s movements, and poor people’s campaigns), claiming that it is now a public health imperative to protect and support them, as with them rests the possibility (and the power?) to push for a system change.

 In fact, history indicates the importance of organized civil society engagement in the achievement of institutional and social change locally, nationally and globally, from legal reforms (eg, the abolition of slavery), to institutional development (eg, environmental protection), to cultural change (eg, gender relations).^4^ The history of people’s movements is also full of acts of resistance that, though limited when considered as such, become relevant when combined in a joint narrative.^5^

 The role of social movements is not only that of building coordinated action that may have the power to bring about change, but also that of growing and nurturing alternative approaches to structuring society and improving health and wellbeing.^6^ Providing a space for different struggles and lived experiences to know, learn from and mutually strengthen one another, social movements have the potential to show today what a different, more caring society may look like tomorrow.

 The People’s Health Movement (PHM), a global network of activist organizations formed in 2000 in response to the failure to achieve Health for All, a goal set in the 1978 Alma Ata Declaration of primary healthcare, is an example in this direction.^7^ From its foundation, PHM activists have argued that “the struggle for health is a political struggle,” one “which challenges the fundamental practices of our society and the trends which shape them.”^8^ Moreover, with its leadership strongly rooted in movements from the Global South, PHM has shown in practice that change can be brought by below particularly if those who suffer most from the current system are engaged in first person in shaping the alternatives.

 For PHM, as for many social movements, the COVID-19 pandemic was a turning point in several ways. As restrictions were imposed on many aspects of social life — including the possibility to organize, show dissent, and practice alternative ways of building society — activists were forced to rethink their practices and move many of them from physical to virtual environments. The availability and accessibility of critical information increased, although linguistic and digital barriers remain, particularly for activists in the Global South. In parallel, during the pandemic the already shrinking space for civil society was, according to reports from human rights organizations and non-governmental organizations, increasingly marked by violence against human rights defenders and representatives of social movements, with activists and socio-cultural workers — including from PHM — subjected to intimidation, bullying, false accusations, unlawful arrests, kidnappings, and murder.^9^ The rise in use of new technologies, a field that saw an exponential growth in the pandemic period, was a powerful way in which increasing governmental control was exerted.^5^

 On the other hand, the pandemic magnified the structural roots of health inequities and made the reasons for health activism even more clear and compelling. PHM saw a rise in engagement at the local and global level, oriented both at supporting those who suffer the most from social injustices amplified by the pandemic, and at striving to bring about the radical changes needed for a more ecojust future (for instance, fighting against vaccine apartheid and the intellectual property regime that makes it possible).^5^

 In this respect, some of the more radical options explored by Ron Labonté in his paper are also being critically debated within PHM. However, this is done in tight connection with the strategies — or theories of change — that may lead from having a vision of how things should change, to making that change happen. Moreover, being aware of the links between colonization processes and knowledge generation, the movement combines visions and practices from different sources, from critical analyses such as the one Ronald Labonté offers, to the ancestral knowledge and wisdom of Indigenous peoples, to the views and practices of the feminist, LGBTQI+ (lesbian, gay, bisexual, transgender, intersex, queer/questioning, asexual and many other terms), and decolonial movements. Building convergence across different social movements and increasing popular participation are key strategies to build the power that is needed for a radical change to happen. Following Ursula Le Guin’s famous quote that “We live in capitalism, its power seems inescapable – but then, so did the divine right of kings. Any human power can be resisted and changed by human beings.”^10^

## Ethical issues

 Not applicable.

## Competing interests

 Author declares that she has no competing interests.
